# IgG4-related disease with interstitial nephritis in a patient with metastatic melanoma following immune checkpoint inhibitor treatment: a case report

**DOI:** 10.1186/s41927-025-00548-1

**Published:** 2025-08-01

**Authors:** Thabuna Sivaprakasam, Prachaya Nitchaikulvatana, Jodi Gedallovich, Jagruti Shah, Matthew Charles Baker

**Affiliations:** 1 Department of Internal Medicine, University of South Dakota Sanford School of Medicine, Sioux Falls, SD USA; 2 Department of Medicine, Division of Immunology and Rheumatology, Stanford University, Palo Alto, CA USA; 3 Department of Pathology, Stanford University, Palo Alto, CA USA; 4 Division of Nuclear Medicine and Molecular Imaging, Department of Radiology, Stanford University, Stanford, CA USA

**Keywords:** Immune checkpoint inhibitors, Immune-related adverse events, IgG4-related disease, Interstitial nephritis, Pembrolizumab, Iplimumab, Nivolumab, Case report

## Abstract

**Background:**

Immune checkpoint inhibitors (ICIs) have become a cornerstone in the treatment of metastatic melanoma. Several case reports have documented IgG4-related disease (IgG4-RD) as an adverse event following ICI therapy. Here we report the first instance of interstitial nephritis associated with IgG4-RD as an immune-related adverse event (irAE) following ICI treatment.

**Case presentation:**

A 71-year-old male with malignant melanoma (BRAF wild-type) initially received one cycle of adjuvant pembrolizumab, followed by four cycles of ipilimumab/nivolumab after the occurrence of lung metastases. Four months later, a follow-up computed tomography (CT) revealed infiltrative masses in the kidneys, along with abnormal mediastinal and hilar lymphadenopathy but his baseline serum creatinine remained stable. A subsequent kidney biopsy showed renal parenchyma with significant interstitial nephritis and an increase in IgG4-positive plasma cells, with no evidence of malignancy. Plasma IgG4 levels were elevated at 294 mg/dL (normal 11–157 mg/dL), and complement C4 level was low at < 8 mg/dL. In addition, the patient had an asymptomatic rise in lipase (105 U/L, normal 7–60 U/L), but had no other findings to suggest pancreatitis. The patient was started on prednisone 40 mg daily with a plan to taper. A follow-up CT scan performed four weeks later showed near-complete resolution of the previously observed mediastinal lymphadenopathy and bilateral infiltrative renal masses.

**Conclusion:**

This represents the first reported case of interstitial nephritis resulting from IgG4-related disease following ICI treatment. Clinicians should consider the potential for IgG4-RD, particularly with associated renal manifestations, in patients undergoing ICI therapy. Early recognition and treatment of this rare side effect can significantly impact the clinical outcome. This case highlights the importance of being vigilant for uncommon and new adverse effects following ICI treatment, especially as the field continues to evolve and new immunotherapies are developed.

**Clinical trial number:**

Not applicable.

**Supplementary Information:**

The online version contains supplementary material available at 10.1186/s41927-025-00548-1.

## Background

Immune checkpoint inhibitors (ICIs) are a group of immunomodulatory therapies that have greatly revolutionized the treatment of various cancers by harnessing the body’s immune system to aid in cancer cell destruction [1, 2]. Immune checkpoints are regulatory systems that prevent overactivation of the immune system; thus, they play an important role in preventing autoimmunity, and inhibiting them can enhance the body’s immune response to tumor cells [1]. While such immune augmentation has improved the response and survival rate in multiple advanced cancers, it can also contribute to unfortunate repercussions in the form of immune-related adverse events (irAEs) [2, 3]. With an often prolonged course, irAEs have an estimated incidence of 15–90% [3]. Immune-related adverse events are organ-specific and can affect virtually any organ system, most commonly the skin, gastrointestinal, and endocrine systems [3]. IgG4-related disease (IgG4-RD)—a fibroinflammatory, immune-mediated disease with multi-organ involvement—is a rarely reported irAE [4]. A few cases of ICI-induced IgG4-RD have been described, with manifestations such as retroperitoneal fibrosis, pancreatitis, and cholangitis [5–7]. Renal disease is relatively rare following ICI, but when it occurs, the most common form is ICI-induced acute kidney injury, with acute tubulointerstitial nephritis being the most common biopsy finding [8].

As the following case demonstrates, our patient developed IgG4-RD with interstitial nephritis on renal biopsy. To our knowledge, this is the first report of a patient developing IgG4-RD interstitial nephritis secondary to ICI therapy.

## Case presentation

A 71-year-old male with stage IV melanoma (BRAF wild-type) and lung metastases received one cycle of adjuvant pembrolizumab, followed by four cycles of ipilimumab/nivolumab. The patient initially presented with two primary melanomas: one on the left shoulder (T2bN0Mx) and another on the left lower leg (T3bN0Mx). He underwent wide local excision (WLE) and sentinel lymph node biopsy (SLNB) for both lesions. Adjuvant therapy was initiated with pembrolizumab, of which he received one cycle. A subsequent staging PET-CT scan revealed metastatic lung nodules. As a result, treatment was escalated to combination immunotherapy with ipilimumab and nivolumab. He demonstrated significant interval improvement of multiple lung nodules following immunotherapy. He had a past medical history of hypertension and gout. Family history was notable for lung cancer in his sister and Crohn’s disease in his brother. He had no history of smoking, alcohol use, or recreational drug use.

Approximately four weeks after completing cycle 4 of ipilimumab and nivolumab, the patient developed diarrhea that progressively worsened, ultimately necessitating hospital admission for further evaluation and management. He had grade 3 immune-related colitis which was treated with a single infusion of infliximab and a prednisone course started at 60 mg tapered over two months, resulting in resolution of the diarrhea. Prednisone was stopped a month before the follow-up imaging. He did not receive maintenance nivolumab therapy due to the colitis and because the follow-up CT imaging demonstrated significant interval improvement in the previously identified pulmonary nodules after 4 cycles of ipilimumab and nivolumab.

**Fig. 1:**
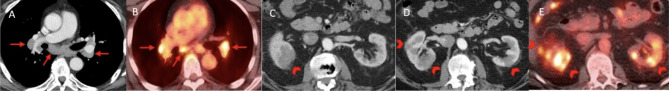
CT and F^18^-FDG-PET/CT images of the chest and abdomen following immunotherapy. Axial contrast enhanced CT images of the chest (**A**) and of the abdomen at the level of the kidneys (**C**, **D**) obtained 4 months following 4 cycles of ipilimumab/nivolumab shows enlarged bilateral hilar and mediastinal lymphadenopathy (red arrows) and bilateral hypodense infiltrative renal cortical masses (red arrow heads). Lung metastases from the melanoma had significantly improved (not shown). Axial fused F^18^-FDG-PET images of the chest (**B**) and abdomen at the level of the kidneys (**E**) post immunotherapy also showed hypermetabolic bilateral hilar and mediastinal lymphadenopathy (red arrows) and hypermetabolic bilateral renal parenchymal masses (red arrowheads)

**Fig. 2:**
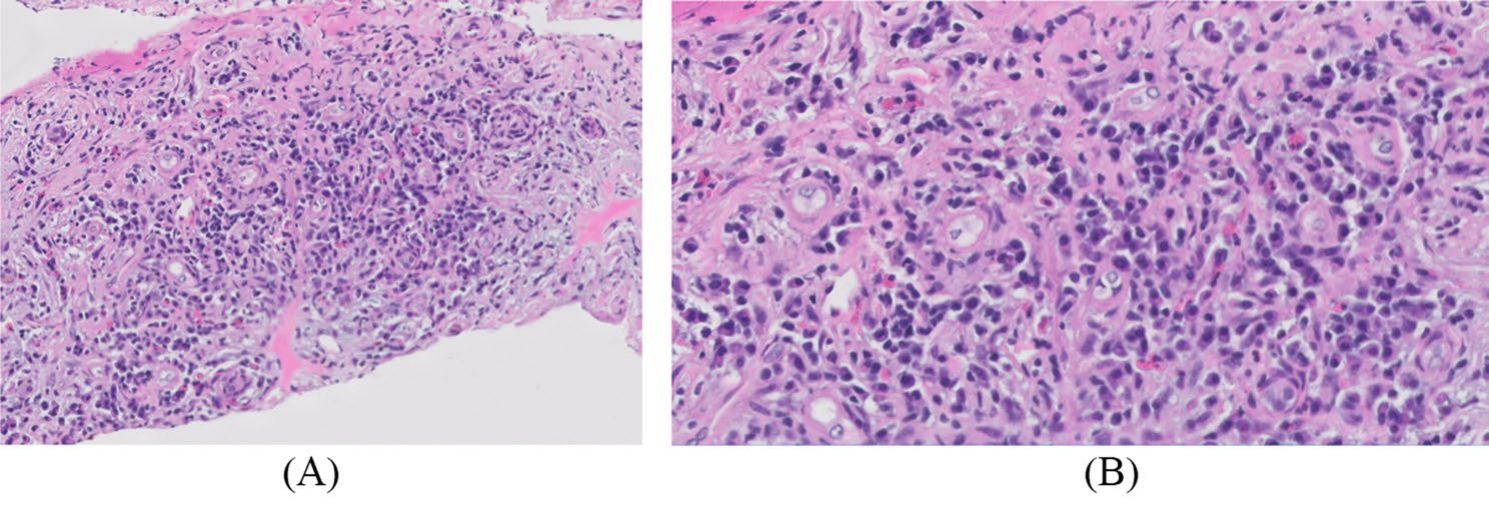
(**A**) H&E-stained renal core biopsy, (**B**) Original magnification x 20. The H&E-stained core biopsy sections show renal parenchyma with marked interstitial inflammation composed predominantly of mature lymphoid cells, plasma cells, and occasional eosinophils (**A**: original magnification, **B**: original magnification X 20)

**Fig. 3:**
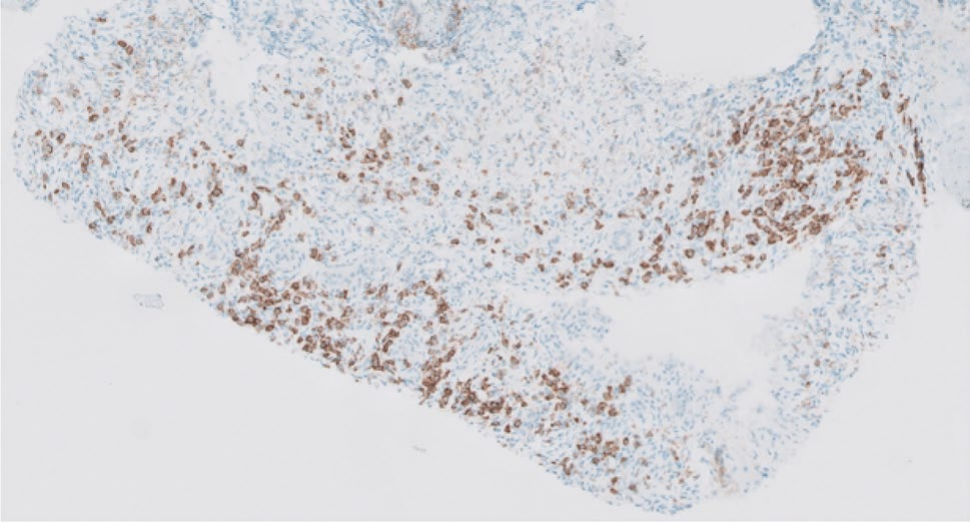
Plasma cell immunohistochemistry. An immunohistochemical stain for CD138 highlights the plasma cell infiltrate

**Fig. 4:**
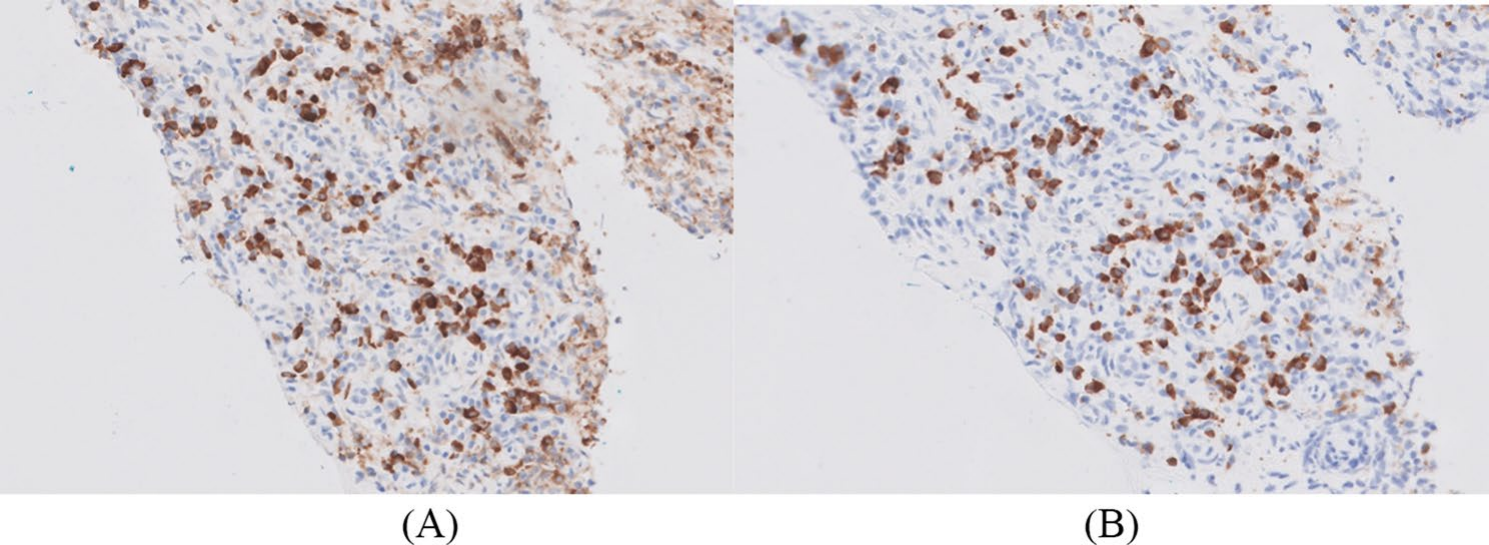
Immunohistochemistry demonstrating (**A**) IgG staining and (**B**) IgG4 staining. Immunohistochemical stains for IgG (**A**) and IgG4 (**B**) demonstrating an abundance of IgG4 + plasma cells with an IgG4:IgG ratio ~ 30%

Four months following the fourth cycle of ipilimumab/nivolumab, a follow-up CT and fluorine18-fluorodeoxyglucose positron emission tomography/computed tomography (F^18^-FDG-PET/CT) (Fig. 1) revealed infiltrative masses in the kidneys, along with abnormal mediastinal and hilar lymphadenopathy. He denied any new symptoms at that time, and the physical exam was unremarkable. A subsequent kidney biopsy (Fig. 2) demonstrated renal parenchyma with significant interstitial nephritis and immunohistochemistry showed an increase in IgG4-positive plasma cells (Figs. 3 and 4) with no evidence of malignancy. The cytologic preparations showed bland renal tubular cells in a background of lymphoplasmacytic inflammatory cells. The core biopsy sections showed renal parenchyma with marked interstitial inflammation composed predominantly of mature lymphoid cells, plasma cells, and occasional eosinophils. CD3 and CD20 immunostains showed the lymphoid cells to be a mixed population of T and B cells. The CD138 positive plasma cells were polytypic by kappa and lambda ISH with an IgG4:IgG ratio ~ 30%. There was an absolute increase in IgG4 plasma cells in multiple foci (98/40x field in area of highest density). These findings were concerning for IgG4-related tubulointerstitial nephritis but were not conclusive.

Biopsy of the mediastinal lymphadenopathy was also considered, but it was determined that the renal biopsy would yield better diagnostic information. After the results of the kidney biopsy showed findings consistent with IgG4-RD, the decision was made to treat and closely monitor the mediastinal lymphadenopathy, as it was deemed likely related to the same pathological process.

The serum IgG4 concentration was elevated at 294 mg/dL (normal 11–157 mg/dL), and complement C4 level was low at < 8 mg/dL. The patient did not exhibit proteinuria, pyuria, or hematuria and his serum creatinine remained stable, within normal range. Lipase was elevated at 105 U/L (normal 7–60 U/L), however no other findings suggestive of pancreatitis were detected.

**Fig. 5:**
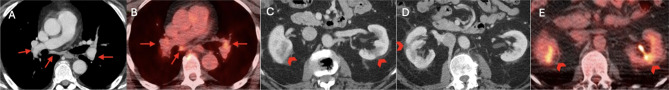
CT and F^18^-FDG-PET/CT images of the chest and abdomen following treatment with prednisone. Axial contrast enhanced CT images of the chest (**A**) and abdomen (**C**, **D**) at the level of the kidneys obtained 4 weeks after initiation of prednisone shows significant decrease in size of the bilateral hilar and mediastinal lymph nodes (red arrows) and bilateral infiltrative renal lesions (red arrowheads). Axial fused F^18^-FDG-PET/CT images of the chest (**B**) and abdomen at the level of the kidneys (**E**) obtained 10 months after steroid initiation also shows significant decrease in the metabolic activity of the thoracic lymph nodes (red arrows) and bilateral renal lesions (red arrowheads)

The patient was initiated on prednisone at a dose of 0.6 mg/kg/day (40 mg/day), with a tapering regimen over four months. A follow-up CT performed four weeks after the initiation of prednisone treatment (Fig. 5) showed near-complete resolution of the previously noted mediastinal lymphadenopathy and infiltrative bilateral renal masses. A subsequent follow-up CT scan four months after completion of prednisone treatment (not shown), demonstrated resolution of thoracic lymphadenopathy, no new lymphadenopathy, as well as multifocal hypoenhancing areas of cortical scarring in both kidneys at the site of previously noted infiltrative masses. F^18^-FDG-PET/CT (Fig. 5) performed ten months after initiation of prednisone also showed resolution of thoracic lymphadenopathy and near-complete resolution of the infiltrative bilateral renal masses.

After glucocorticoid treatment, the IgG4 concentration returned to normal. The patient reported in outpatient follow-up that this medical journey had been overwhelming, but that he was incredibly grateful for the care he received.

## Discussion

Traditionally, cancer treatment has included surgery, radiation, and chemotherapy, but recently the emergence of cancer immunotherapy has revolutionized the field [3]. By promoting inhibition of immune checkpoints and subsequent enhancement of T-cell-mediated anti-tumor response, immunotherapy has shown significant benefit in many dermatologic, hematologic, urogenital, gastrointestinal, and pulmonary malignancies [8]. The commonly used FDA-approved ICIs include nivolumab and pembrolizumab (PD-1 inhibitors), ipilimumab (CTLA-4 inhibitor), durvalumab and atezolizumab (PD-L1 inhibitors), and relatlimab (LAG-3 inhibitor) [3, 4].

While the ICI-mediated T cell response is helpful for tumor destruction, the resulting disruption of immune self-tolerance can lead to immune-related adverse events (irAEs) [2, 3]. Immune-related adverse events can potentially affect any organ system with the most common manifestations observed in the gastrointestinal tract, endocrine system, skin, eyes, and musculoskeletal systems [9]. The incidence of irAEs is highly variable, dependent on the ICI agent used and the risk markedly increases with combination regimens compared to monotherapy [3, 9]. These adverse effects are mostly mild-moderate in severity and reversible with appropriate management [3] but certain manifestations such as pneumonitis, myocarditis, and nephritis, can be fatal [9].

Our patient is a 71-year-old male with BRAF wild-type metastatic melanoma who was diagnosed with a rare irAE after being treated with PD-1 and CTLA-4 immunotherapy. He was incidentally found to have infiltrative masses in the kidneys, along with mediastinal and hilar lymphadenopathy with no significant clinical findings. The kidney biopsy was crucial for diagnosis, showing significant interstitial nephritis with IgG4-positive plasma cells which alongside the elevated serum IgG4 concentration and low complement levels helped elucidate the final diagnosis of IgG4-related disease [4].

While renal irAEs secondary to ICIs are less common, they are increasingly observed; the most common renal pathology observed is tubulointerstitial nephritis [8, 10]. These renal irAEs were commonly associated with risk factors, such as pre-existing renal disease, concomitant PPI use, and the use of combination immunotherapy regimens [11]. However, the ICI-induced interstitial nephritis observed in our patient was secondary to IgG4-RD, which is a rare entity not previously reported.

IgG4-related disease is an immune-mediated disease that causes fibroinflammatory lesions, diffuse organ enlargement, or wall thickening, with a reported national incidence of 0.78–1.39 per 100,000 person-years [4, 12]. The American College of Rheumatology/European League Against Rheumatism (ACR/EULAR) published classification criteria for IgG4-related disease in 2019, which can be helpful when the diagnosis is being considered [4]. After satisfying the inclusion and exclusion criteria, a score of ≥ 20 points classifies a patient as having IgG4-related disease [4]. Our case satisfies the entry criteria with characteristic radiologic/pathologic involvement of a typical organ. Based on the investigations conducted, no exclusion criteria were met, and a total of 25 points were accrued (serum IgG4 level 2-5x upper limit of normal [+ 6], histopathology showing dense lymphocytic infiltrate [+ 4], immunostaining with IgG4+/IgG ~ 30% and IgG4 cells/HPF > 10 [+ 7] and renal pelvis thickening/soft tissue [+ 8]), thus confirming a diagnosis of IgG4-related disease. In addition, the clinical response to treatment with glucocorticoids further strengthened the diagnosis of IgG4-related disease.

Although rare, IgG4-RD secondary to ICI therapy has been reported in the literature involving organs such as pancreas, bile ducts, pituitary, retroperitoneum, and pleura [5–7, 13, 14]. In patients without exposure to ICIs, IgG4-RD is known to affect the renal system, manifesting commonly as tubulointerstitial nephritis or membranous nephropathy, affecting 15% of the IgG4-RD patients [15]. To our knowledge, this is the first case of IgG4-RD induced by ICIs manifesting with renal involvement. Though spontaneous IgG4-RD is possible, the timeline of disease emerging after the ICI therapy and the absence of any pertinent signs or symptoms before the treatment suggests this is unlikely. A direct association between this patient’s malignancy and IgG4-RD is another consideration but malignancy usually occurs after IgG4-RD diagnosis [16], and there is no evidence to date suggesting IgG4-RD can be caused by cancer.

The ability of both ICI therapy and IgG4-RD to individually induce interstitial nephritis can be attributed to their overlapping pathogenic mechanisms. Some of the proposed mechanisms for the development of irAEs include cytotoxic T-cell activation and autoantibody production by B cells propagated by the ICIs, which leads to destruction of healthy tissue [9]. Though the pathogenesis of IgG4-RD is unclear, T cells and B cells play central roles [12]. Two important T cell subsets have been identified in affected tissues: (1) type 2 T follicular helper cells producing cytokines such as IL-4 and IL-13, which support class-switching to IgG4 and IgE producing B cells and (2) CD4 + cytotoxic T lymphocytes, which promote tissue apoptosis and fibrosis [12]. This mechanistic overlap between the pathogenesis and cell populations of ICI-induced irAEs and IgG4-RD may also explain how ICI-induced IgG4-RD can occur. The ICIs used in our patient function by blocking the co-inhibitory PD-1/PDL-1 and CTLA-4/B-7 pathways on the surface of various T cell populations, thus enhancing T cell activation [2]. Such augmented activation and expansion of the T follicular helper cells (causing IgG4 + B cell expansion) and cytotoxic T cells (causing fibrosis) could explain the pathogenesis behind the IgG4-predominant interstitial nephritis in our patient.

Finally, systemic steroids are valuable immunosuppressive options used to provide clinical improvement in both irAEs and IgG4-RD [2, 12]. Our patient had an excellent response to prednisone, with a near-complete resolution of the previously observed mediastinal lymphadenopathy and infiltrative renal masses. This case therefore underscores the importance of early recognition of renal irAEs, with renal biopsy being crucial for diagnosis [2, 17]. Such early recognition can help initiate the appropriate treatment such as corticosteroids, which will significantly improve clinical outcomes.

## Conclusion

Though renal manifestations are associated independently with IgG4-RD and ICI therapy, our case highlights the rare convergence of these entities as ICI-induced renal IgG4-RD. The expanding spectrum of organ involvement in ICI-induced IgG4-RD as demonstrated in our patient and other case reports [5–7, 13, 14] highlights the need for a high index of suspicion in atypical presentations during ICI therapy. Including ICI-induced IgG4-RD in the differential diagnosis will enable early identification, appropriate management, and optimal outcomes.

## Supplementary Information

Below is the link to the electronic supplementary material.

Supplementary Material 1

## Data Availability

No datasets were generated or analysed during the current study.

## Abbreviations

ICI: Immune Checkpoint Inhibitors
IgG4-RD: IgG4-Related Disease
irAEs: Immune-related Adverse Effects
F18-FDG PET/CT: Fluorine18-Fluorodeoxyglucose Positron Emission Tomography/Computed Tomography
PD-1: Programmed Death-1
PD-L1: Programmed Death-Ligand 1
CTLA-4: Cytotoxic T Lymphocyte-associated Antigen-4
LAG-3: Lymphocyte-Activation Gene-3
AKI: Acute Kidney Injury
ACR/EULAR: American College of Rheumatology/European League Against Rheumatism
