# Supplementary material for: IgG4-related disease with interstitial nephritis in a patient with metastatic melanoma following immune checkpoint inhibitor treatment: a case report
Source: BMC Rheumatol. 2025 Aug 1;9:95. doi: 10.1186/s41927-025-00548-1 (PMC12315461; doi:10.1186/s41927-025-00548-1)
Supplement: Supplementary file 1 — Supplementary Material 1 [file 41927_2025_548_MOESM1_ESM.pdf]

CARE case report guidelines

CARE Checklist of information to include when writing a case report

(cc) BY-NC-ND

Topic	Item	Checklist item description	Reported on Line
Title	1	The diagnosis or intervention of primary focus followed by the words "case report"	1,2
Key Words	2	2 to 5 key words that identify diagnoses or interventions in this case report, including "case report"	56,57
Abstract (no references)	3a	Introduction: What is unique about this case and what does it add to the scientific literature?	59,79
	3b	Main symptoms and/or important clinical findings	96,97
	3c	The main diagnoses, therapeutic interventions, and outcomes	97-100, 128-137
	3d	Conclusion—What is the main "take-away" lesson(s) from this case?	261-273
Introduction	4	One or two paragraphs summarizing why this case is unique (**may include references**)	31-35, 70-79
Patient Information	5a	De-identified patient specific information.	81-86
	5b	Primary concerns and symptoms of the patient.	96,97
	5c	Medical, family, and psycho-social history including relevant genetic information	84-86
	5d	Relevant past interventions with outcomes	88-96
Clinical Findings	6	Describe significant physical examination (PE) and important clinical findings.	96,97
Timeline	7	Historical and current information from this episode of care organized as a timeline	93-146
Diagnostic Assessment	8a	Diagnostic testing (such as PE, laboratory testing, imaging, surveys).	93-127
	8b	Diagnostic challenges (such as access to testing, financial, or cultural)	N/A
	8c	Diagnosis (including other diagnoses considered)	212-221
	8d	Prognosis (such as staging in oncology) where applicable	81
Therapeutic Intervention	9a	Types of therapeutic intervention (such as pharmacologic, surgical, preventive, self-care)	128,129
	9b	Administration of therapeutic intervention (such as dosage, strength, duration)	128,129
	9c	Changes in therapeutic intervention (with rationale)	N/A
Follow-up and Outcomes	10a	Clinician and patient-assessed outcomes (if available)	129-137, 146-148
	10b	Important follow-up diagnostic and other test results	129-137, 146
	10c	Intervention adherence and tolerability (How was this assessed?)	128,129
	10d	Adverse and unanticipated events	N/A
Discussion	11a	A scientific discussion of the strengths AND limitations associated with this case report	N/A
	11b	Discussion of the relevant medical literature **with references**.	151-180, 194-207, 223-259
	11c	The scientific rationale for any conclusions (including assessment of possible causes)	212-221, 229-252
	11d	The primary "take-away" lessons of this case report (without references) in a one paragraph conclusion	269-273
Patient Perspective	12	The patient should share their perspective in one to two paragraphs on the treatment(s) they received	146-148
Informed Consent	13	Did the patient give informed consent? Please provide if requested	Yes ☑ No ☐
